# Utility of R_2_* Obtained from T_2_*-Weighted Imaging in Differentiating Hepatocellular Carcinomas from Cavernous Hemangiomas of the Liver

**DOI:** 10.1371/journal.pone.0091751

**Published:** 2014-03-14

**Authors:** Meiyu Sun, Sheng Wang, Qingwei Song, Zhiyuan Wang, Heqing Wang, Dianxiu Ning, Bin Xu, Qiang Wei, Ailian Liu

**Affiliations:** 1 Department of Radiology, the First Affiliated Hospital, Dalian Medical University, Dalian, Liaoning, China; 2 Department of CT and MRI, Fuxin Mineral Hospital, Fuxin, Liaoning, China; 3 Department of Ultrasound, Hunan Provincial Tumor Hospital and Affiliated Tumor Hospital of Xiangya Medical School, Central South University, Changsha, Hunan, China; Wayne State University, United States of America

## Abstract

**Purpose:**

To evaluate the feasibility of applying R_2_* values to differentiate hepatocellular carcinomas (HCC) from cavernous hemangiomas of the liver (CHL).

**Materials and Methods:**

This retrospective study was approved by the participating Institutional Review Board and written informed consent for all subjects were obtained. Seventy-three patients with 79 pathologically identified HCCs and 65 patients with 91 clinically or pathologically identified CHLs were enrolled in this study. All subjects underwent a breath-hold multi-echo T_2_* weighted MR imaging on a 1.5T clinical MR scanner. R_2_* values from HCC and CHL groups were compared using the Mann-Whitney non-parametric *U* test. A cut-off value of R_2_* was evaluated with receiver operator characteristic (ROC) analysis.

**Results:**

The mean R_2_* value was 23.32±12.23 Hz (95% confidence interval [CI]: 20.58 Hz, 26.06 Hz) for the HCC group, and 3.66±2.37 Hz (95% CI: 3.17 Hz, 4.15 Hz) for the CHL group. The mean R_2_* value for HCC was significantly higher than that of CHL (*p*<0.001). A threshold of 9.48 Hz for the minimum R_2_* value in the diagnosis of HCC resulted in a sensitivity of 96.20% (76 out of 79 patients), and a specificity of 97.80% (89 out of 91 patients). The positive predictive value (PPV), negative predictive value (NPV) and diagnostic accuracy for HCC were 97.44% (76 out of 78 patients), 96.74% (89 out of 92 patients) and 97.06% (165 out of 170 patients), respectively. The AUC for differentiation between these two groups was 0.994 (95% CI: 0.980, 1.000).

**Conclusions:**

R_2_* is a significant MRI biomarker to differentiate HCC from CHL with satisfying sensitivity and specificity.

## Introduction

Hepatocellular carcinoma (HCC) is the most common type of primary malignant hepatic tumor, which is the third leading cause of cancer-related death worldwide [1]. Cavernous hemangioma of the liver (CHL) is the most frequently diagnosed benign hepatic tumor, developing in 0.4–20% of the population [2]. Treatment for CHL is markedly different than HCC [3],[4]. Characterization of CHL and HCC using ultrasonography (US) and contrast-enhanced computed tomography (CT) is well established and routinely applied in liver lesion diagnosis [5]–[7]. Both qualitative and quantitative magnetic resonance imaging (MRI) methods are also used for the diagnosis of liver diseases [8]–[13]. Subjective qualitative analysis depends on interpreters’ experience and expertise, thereby potentially limiting its diagnostic accuracy and reproducibility. Additionally, administration of gadolinium contrast agents is required for this type of subjective interpretation. Several noninvasive, quantitative MR methods for differentiating malignant tumors from benign hepatic lesions have also been proposed, including calculated T_2_ relaxation times [9]–[11] and apparent diffusion coefficient (ADC) derived from diffusion weighted imaging [13]. Despite the availability of these non-invasive methods mentioned above, the considerable overlaps, sequence dependent results, and long acquisition times have prevented their wide use in routine clinical applications. Therefore, to circumvent these limitations, the current study proposes the establishment of an accurate, fast and robust quantitative method using non-invasive MRI.

R_2_* (1/T_2_* in 1/s) is sensitive to both micro and macro vasculature due to susceptibility alteration [14]. A prior study has already demonstrated the feasibility of using R_2_* values to predict the characteristic micro vascular invasion of HCC using a blood oxygen level-dependent (BOLD) test [14]. Several recent studies have also suggested that multi-echo T_2_*-weighted imaging might have the potential to identify HCC [15]–[17]. However, in current literature, there is no study focused on the usefulness of R_2_* in differentiating HCC from CHL. Therefore, the purpose of this study is to investigate the feasibility of using R_2_* values from multi-echo T_2_*-weighted imaging to differentiate HCC and CHL.

## Materials and Methods

### Patients

This study was approved by the Institutional Review Board of the First Affiliated Hospital of Dalian Medical University and all subjects signed a written informed consent. From November 2011 to February 2013, 138 consecutive HCC and CHL patients were enrolled in this study. The study excluded HCC patients (n = 465) with prior treatment including antineoplastic chemotherapy, trans-arterial chemoembolization, radiofrequency ablation, or chemo radiation. An additional 14 HCC and 12 CHL patients were excluded due to severe motion artifact from an unsuccessful breath-hold or cardiac and aortic pulsation. Seventy-three patients (mean age = 60.10 years ±11.32(SD); 60M: 13F) with 79 pathologically identified HCC and 65 patients (mean age = 47.74 years ±9.03(SD); 26M: 39F) with 91 clinically or pathologically identified CHL were enrolled in this study. None of the subjects enrolled had hemochromatosis, severe hepatic steatosis, or intravenous administration of super-paramagnetic iron oxide particles before the MRI examination.

For the 73 subjects with HCCs (79 lesions), all had histopathological confirmation after biopsy (n = 15, including two patients with two lesions) or surgical resection (n = 58, including 57 patients with solitary HCC lesion and one patient with five HCC nodules). The interval between the date of the MRI study and sample collection for histopathological confirmation ranged from 4–7 days (average 5.5 days±1.0 (SD)). Among the 65 patients with CHL, 26 patients carried diagnosed cavernous hemangiomas for more than two years before the MR study and had no changes in morphology and size during this period. The diagnosis of CHL was established by means of observed hyper intensity on T_2_-weighted images and slightly irregular or globular peripheral enhancement with gradual filling in the center of the lesion on delayed images in CT and/or MRI with intravenous administration of contrast material [18]. At the time they had their MR examinations, confirmed diagnosis of CHL for another 37 patients was made based on typical patterns of enhancement on dynamic MRI examination and the absence of change in morphology and size of the lesions during a follow-up period of more than six months after the imaging studies (mean: 12 months; range: 6–16 months). The remaining two patients with CHL had histopathological confirmation after hepatectomy.

### MR Imaging

Each patient was instructed to hold his or her breath in a fixed position on all breath-hold sequences. All examinations were performed on a 1.5T (40 mT/m) MR scanner (Signa, HDxt, General Electric [GE] healthcare, USA) with an 8-channel phased array coil, adequately positioned to cover the upper abdomen of the patient lying in the supine position. All patients underwent a routine MRI examination initially for the upper abdomen which included transverse T_2_-weighted imaging and dynamic T_1_-weighted imaging. T_2_-weighted fat-suppressed images were obtained with a respiratory-triggered fast spin echo (T_2_WFSE) sequence. The parameters were: repetition time (TR) = 6000 ms, echo time (TE) = 102 ms, bandwidth = 62.5 Hz/pixel, NEX = 3, acquisition matrix = 288×224, field of view (FOV) = 380 mm×380 mm, slice thickness = 6 mm, gap = 1.5 mm, and scan time three minutes. Dynamic T_1_-weighted MRI was obtained by using a 3D T1-weighted fat-suppressed spoiled gradient echo sequence utilizing the manufacturer’s liver acquisition volume acceleration (LAVA) technique before and after injection of gadopentetate dimeglumine (Magnevist, Schering, Guangzhou, China) at a dose of 0.1 mmol per kilogram of body weight mass in the distal part of a connecting line into an antecubital vein at a rate of 2 ml/s using a power injector (Meorao, Spectris, USA). Acquisition parameters were as follows: TR = 3.7 ms, TE = 1.8 ms, inversion time = 7 ms, flip angle = 20°, parallel imaging (ASSET: array spatial sensitivity encoding technique, GE) acceleration factor = 2, bandwidth = 62.5 Hz/pixel, NEX = 0.7, acquisition matrix = 272×180, FOV = 380 mm×380 mm, slice thickness = 4.4 mm, gap = −2.2 mm, and scan time was about 17 s per phase. Multi-echo T_2_*-weighted was performed prior to dynamic T_1_-weighted imaging, the parameters were: TR = 16.4 ms; TE = 2.1, 5.0, 7.9, 10.9, 13.8 ms; flip angle = 20°; ASSET acceleration factor = 2: bandwidth = 62.5 Hz/pixel; NEX = 0.67; acquisition matrix = 256×192; FOV = 380 mm×380 mm; slice thickness = 2 mm; and 21 s scan time for one breath-hold acquisition.

### MRI Evaluation

Two radiologists (Liu AL with 20 years and Sun MY with 10 years of experience in reading hepatic MRI images) reviewed the MRI images using a PACS work station (Daijia healthcare, Shanghai, China). They were both blinded to the original MRI reports and did not know the proportion of each type of lesion in the study population. MRI diagnosis of each patient was obtained in conference by two observers informed with necessary clinical history and previous images.

### R_2_* Measurement

The multi-echo T_2_*-weighed images were transferred to an ADW4.4 workstation (Sun Microsystems, Santa Clara, Calif, USA) for post-processing. To generate the R_2_* map, the logarithms of the pixel values, S, were fitted to a linear function using regression analysis, according to the equation S(TE) = S(0)⋅exp (−TE⋅R_2_*). All measurements were performed by a radiologist (WS with 5 years of experience in reading liver MRI images) who was blinded to the diagnosis of the subjects. The largest possible ROI, which represented the signal intensity of the tumor, was manually outlined in a homogeneous area on the R_2_* map. The contrast and morphologic characteristics in the three phases of contrast-enhanced T_1_-weighted imaging and T_2_-weighted imaging were used to guide ROI placement in order to avoid visible vasculature, biliary structures, hemorrhage, suspected calcification and necrosis. The areas which were severely affected by artifacts caused by the tissue-air interface, pulsations of the heart, or abdominal aorta were not included. To demonstrate the reproducibility of the results, all HCC and CHL lesions were measured three times with the same method by varying placement of the ROIs and the final R_2_* were taken as the averaged value for the three times. Furthermore, the R_2_* values were measured repeatedly on two sessions with a one month interval by the abovementioned observer working in consensus to test the intraobserver concordance. The R_2_* value and associated SDs for each region of interest were calculated on a pixel-by-pixel basis.

### Statistical Analysis

SPSS 17.0 software for Windows (SPSS Inc., Chicago, Illinois, USA) was used for statistical analysis. The size difference of the HCC and CHL groups were searched for using the student’s *t*-test. Mean R_2_* values as well as standard deviations, medians, and ranges were calculated for the HCC and CHL groups. The precision of R_2_* measurements were tested by the reliability coefficient (*r_c_*). The *r_c_* values larger than 0.85, between 0.50 and 0.85, and less than 0.50 indicated good concordance, moderate concordance, and poor concordance, respectively. The distributions of R_2_* obtained for HCCs and CHLs were displayed in box plots. The Mann-Whitney non-parametric *U*-test was used to compare the mean R_2_* value in the HCC and CHL groups. A probability value (*p*) less than 0.05 was considered statistically significant. Receive operating characteristic (ROC) curve analysis was performed to identify the optimum cutoff value which maximized the average of sensitivity and specificity, with HCC defined as test positive and CHL as test negative. Sensitivity, specificity, positive predictive value (PPV), negative predictive value (NPV) and accuracy of diagnosis were subsequently calculated and recorded. The diagnosis performance of R_2_* values for differentiating HCCs from CHLs was evaluated with the calculation of the area under the curve (AUC).

## Results

### Intraobserver Concordance of the R_2_* Value Measurements

In this cohort, the mean R_2_* value of the HCC group was 23.32±12.23 Hz (range, 5.29–73.74) for initial measurements and 21.78±11.25 Hz (range, 5.16–72.93) for the repeated measurements. The resulting *r_c_* value was 0.93, which was considered acceptable. The mean R_2_* value of the CHL group was 3.66±2.37 Hz (range, 0.22–10.88) for initial measurements and 3.69±2.25 Hz (range, 0.21–10.97) for the repeated measurements, resulting in an *r_c_* value equal to 0.96 which was acceptable as well. Therefore, the intraobserver concordance of the R_2_* value measurements was proven sufficient and the first measurements were used as the final R_2_* values.

### Comparison of R_2_* Values between HCC and CHL Groups

**Figure 1:**
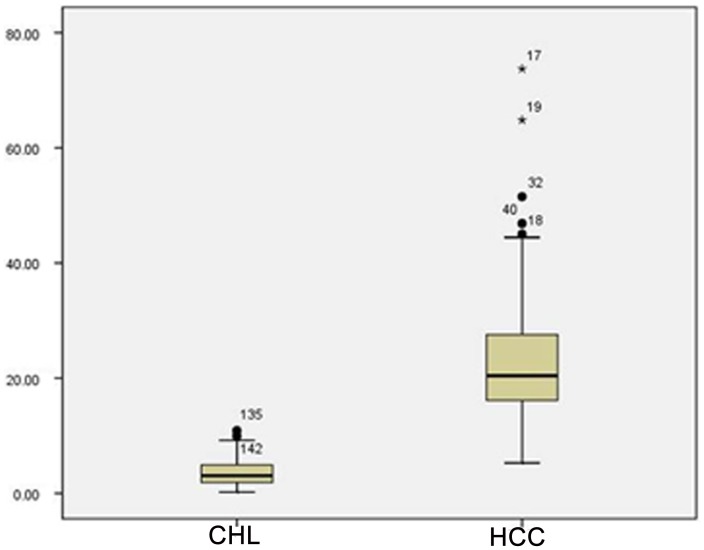
Box plots of R_2_* value of HCCs and CHLs. R_2_* value is differed significantly between HCCs and CHLs. Boxes stretch across interquartile range (IR), from lower quartile (Q1) to upper quartile (Q2); whiskers show the smallest data point that is greater than [Q1–1.5IR] and the largest data point that is smaller than [Q2+1.5IR]. The horizontal line through each box represents the median value. The dot indicates any outlier with its code number.

**Figure 2:**
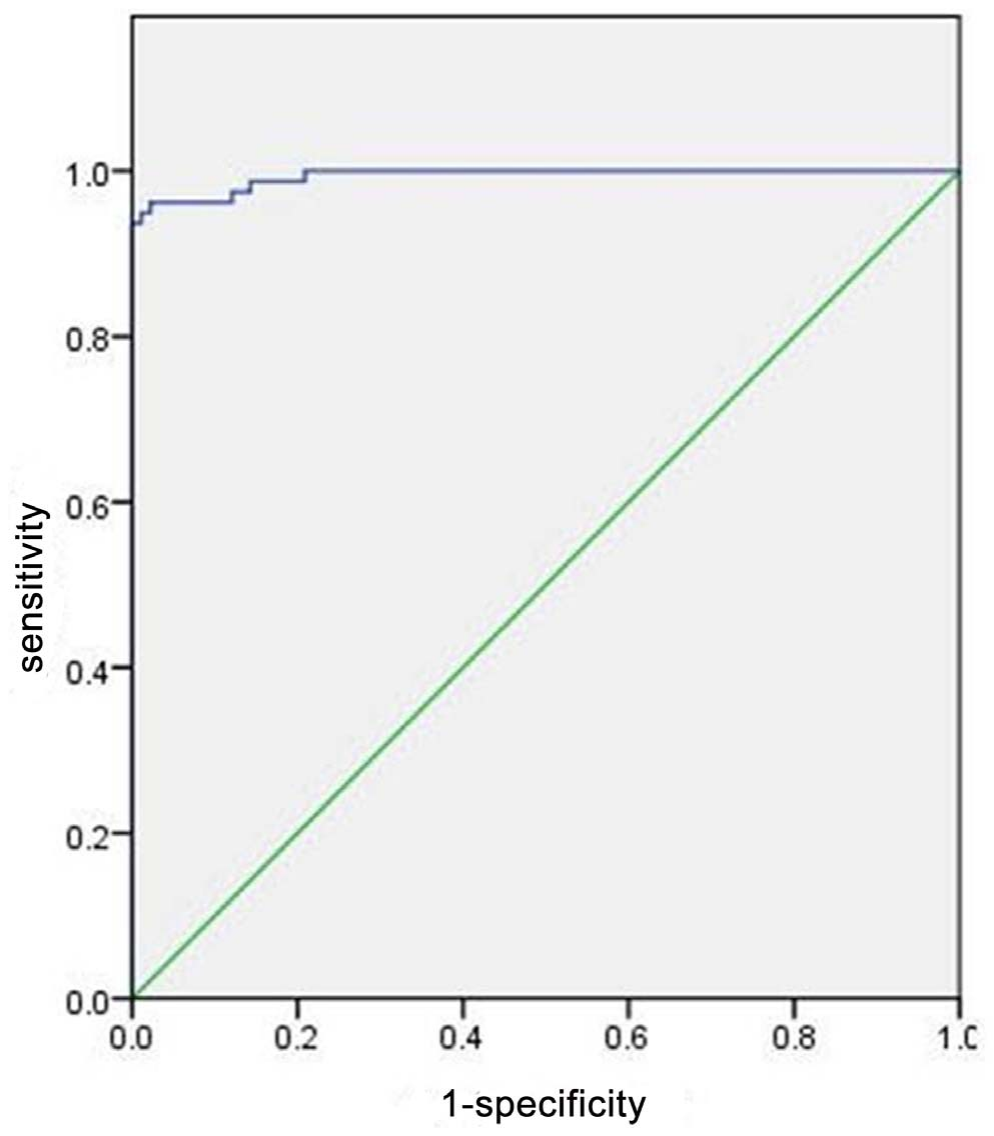
ROC curve for the diagnosis of HCCs. Area under ROC curve is 0.994 (95% CI: 0.980, 1.000).

**Figure 3:**
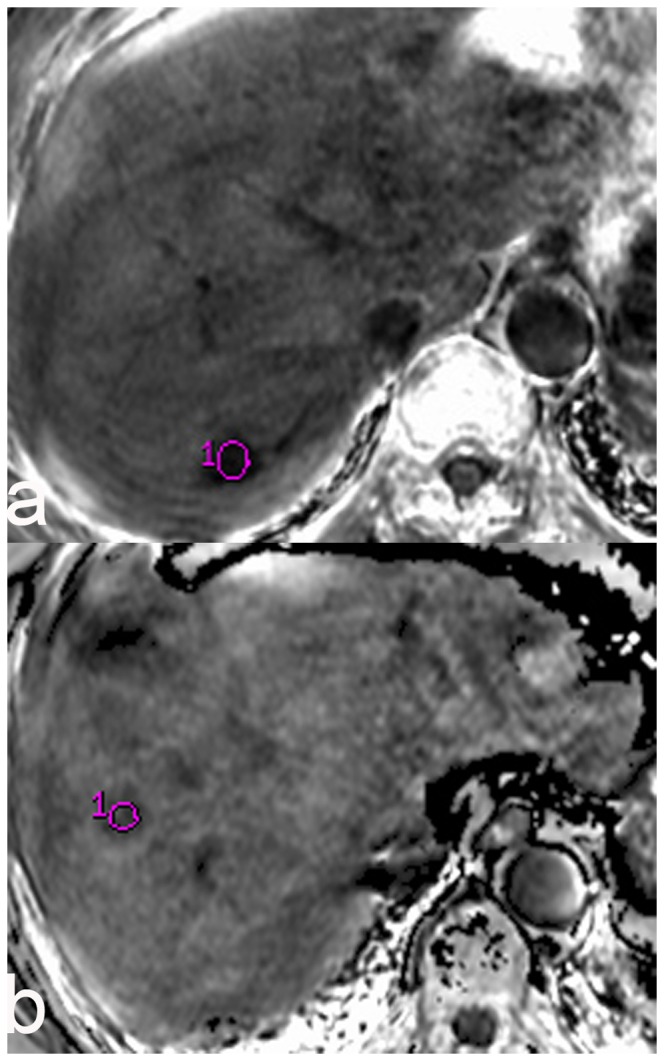
R_2_* images of CHL and HCC. a. A CHL R_2_* image in a 51-year-old female. Transverse R_2_* map shows a well-marginated lesion at segment VII of the liver with a mean R_2_* value of 7.77 Hz. b. A HCC R_2_* image in a 59-year-old female. Transverse R_2_* map shows a well-marginated lesion at segment VIII of the liver with a mean R_2_* value of 14.41 Hz.

A total of 138 subjects (170 lesions) were enrolled, with a mean lesion size of 3.78±2.10 cm (range from 0.89 cm to 9.34 cm). For the 79 HCC lesions, the mean size was 4.26±2.10 cm (range from 1.1 cm to 9.1 cm), and the mean R_2_* value was 23.32±12.23 Hz (95% CI: 20.58 Hz, 26.06 Hz). For the 91 CHL lesions, the mean size was 3.37±2.03 cm (range from 0.89 cm to 9.34 cm) and the mean R_2_* value was 3.66±2.37 Hz (95% CI: 3.17 Hz, 4.15 Hz). No significant differences in lesion size were found between the two groups of patients (students’ *t*-test, *p*<0.001). Table 1 lists the R_2_* results of the Mann-Whitney non-parametric *U*-test. The mean R_2_* value of HCCs (Hz) was significantly higher than that of CHLs (*p*<0.001). The R_2_* value of HCCs and CHLs were plotted in box and whisker format respectively on figure 1. The boxes showed the distance between the first and the third quartiles, with the median marked as a line, and the “whiskers” indicating the range. The ROC curve was shown in figure 2. The optimal R_2_* value calculated from the ROC curve was 9.48 Hz, which resulted in a sensitivity of 96.20% (76/79), specificity of 97.80% (89/91), PPV of 97.44% (76/78), NPV of 96.74% (89/92), and a diagnosis accuracy of 97.06% (165/170). Figure 3 shows the typical appearance of CHL and HCC on axial R_2_* maps. Overlapping of these two types of hepatic lesions was found and five lesions (5/170, 2.94%) involved in this study. Applying the R_2_* measurement threshold of 9.48 Hz as a parameter, 2 out of 91 CHLs (2/91, 2.20%) were erroneously considered as HCCs, whereas three HCCs (3/79, 3.80%) were erroneously considered as CHLs.

**Table 1:** R_2_* value of HCC and CHL groups.

R_2_*(Hz)	mean±SD	Median(*q_1_;q_3_*)	Range	Z	*P*
**HCC**	23.32±12.23	20.41(16.05;27.59)	5.29–73.74	−11.807	0.000
**CHL**	3.66±2.37	3.05(1.78;4.98)	0.22–10.88	−11.807	0.000

Note. SD indicates standard deviation. *q_1_* indicates first quartile. *q_3_* indicated third quartile. Significance was searched for using the Mann-Whitney non-parametric *U*-test.

## Discussion

Treatment options and the overall management for HCC and CHL are different. Hepatic resection or transplantation are the best treatment options currently available for HCCs [4], and interventional therapies such as transcatheter arterial chemoembolization (TACE), radio-frequency ablation and microwave ablation are the alternative steps for those not eligible for surgical treatment [19]. In contrast, CHL does not require surgical resection, with the exception of large symptomatic lesions, due to the low rate of complications [3]. Percutaneous liver biopsy is currently considered as the gold standard for assessing hepatic neoplasm; however, this invasive method is costly and somewhat risky, thereby limiting its availability as an option for many patients [4]. A correct diagnosis of HCC and CHL based on non-invasive or minimally-invasive imaging would be of great benefit in guiding subsequent treatment planning.

This study demonstrates that R_2_* is a valuable tool for differentiating HCC from CHL. Using 9.48 Hz as the threshold, we found a 96.20% sensitivity, a 97.80% specificity, a 97.44% PPV, a 96.74% NPV, and a diagnosis accuracy of 97.06%. This result shows better differentiation than all previously reported studies in literature. Moreover, with a total acquisition time of 21 seconds, this method is much faster than all other reported methods in similar studies. Chan *et al*. [10] used moderate T_2_-weighted imaging with a scan time of more than 2.5 minutes to differentiate benign and malignant hepatic lesions. They achieved a 77% specificity and 88% PPV with a T_2_ relaxation time threshold of <112 ms being indicative of malignant lesion. Soyer *et al* [13] differentiated CHL from untreated malignant hepatic neoplasms with free-breathing diffusion-weighted MRI with a scan time around 2 minutes. With two independent readers, they reported a sensitivity of 65.7%∼100%, a specificity of 85.7%∼100% and an accuracy of 82.9%∼94.3% depending on which threshold was applied to the ADC values. It is noteworthy that in the discussed study, two subjects underwent unnecessary surgery because their hepatic lesions were unfortunately misdiagnosed as HCC on conventional MRI. The R_2_* value of these two cases was 7.77 and 9.19 Hz respectively, which are smaller than our threshold value of 9.48 Hz. In other words, they would have been correctly diagnosed if the usefulness of R_2_* was discovered earlier. In the present study, 2 out of 91 CHLs were also erroneously considered as HCCs, however, these two CHLs were characterized by typical imaging features on routine MRI. This study supports that R_2_* values could potentially provide an effective quantitative parameter to help differentiate difficult or confusing HCC and CHL cases.

This study indicates that the mean R_2_* value of HCC is significantly higher than that of CHLs. The exact mechanism behind this observation is unknown. A possible explanation is the lack of sufficient oxygenation in HCC. Hypoxia is common within a tumor micro environment due to the imbalance between oxygen supply from abnormal tumor vasculature and the high resource demand necessary for the rapid proliferation of tumor cells [19]–[21]. It is well known that R_2_* is related to the oxygenation state of hemoglobin [14], [15]. Blood in the microvasculature of HCC is lower in oxygenation and, accordingly, the deoxyhemoglobin concentration is higher which results in an increase in R_2_* values. CHL, on the other hand, involves a vascular malformation of the liver arterioles. It has been observed that the veins which drain the CHL, the peripheral branches of the portal vein, have 85% oxyhemoglobin saturation [22], [23]. This increased concentration of oxyhemoglobin in CHLs can therefore result in a decrease in R_2_* values. Furthermore, intra-tumor hemorrhage in HCCs, which is rarely encountered in CHLs, may also contribute to its increased R_2_* value. Although R_2_* values were obtained from ROIs which did not include any visible hemorrhage on T_1_WI and T_2_WI in HCC, it does not mean that the ROI does not include micro-hemorrhage As demonstrated in recent studies [24], [25], more intra-tumorous hemorrhage, especially micro-hemorrhage, were detected in HCC with susceptibility weighted imaging than with conventional T_1_W and T_2_W images. When there was internal spontaneous micro-hemorrhages, the extravasated hemoglobin within the tumor was converted to deoxyhemoglobin or hemosiderin. These paramagnetic substances could lead to local magnetic field inhomogeneity, resulting in the dephasing of protons and an increase in R_2_* values [26], [27].

This study has some limitations. The major weakness is the relatively small number of patients with histopathological confirmation of CHLs. This study mainly characterized CHL based on imaging characteristics and stability in size over time. To circumvent this limitation, only patients with histopathologically or clinically proven CHL as determined by two experienced radiologists after at least six months of CT and/or MRI follow-ups were selected.

In conclusion, this study demonstrates that using R_2_* measurements from multi-echo T_2_*-weighted imaging appears to be a simple yet robust method to differentiate HCC from CHL with satisfying sensitivity and specificity.
